# The ability to appropriately distinguish throws for different target positions

**DOI:** 10.3389/fspor.2023.1250938

**Published:** 2023-09-06

**Authors:** Ayane Kusafuka, Rintaro Yamamoto, Taishi Okegawa, Kazutoshi Kudo

**Affiliations:** ^1^ Department of Intermedia Art and Science, Faculty of Science and Engineering, Waseda University, Tokyo, Tokyo, Japan; ^2^ Japan Society for the Promotion of Science (JSPS), Japan; ^3^ Department of Life Science, Graduate School of Arts and Sciences, The University of Tokyo, Tokyo, Japan

**Keywords:** pitching, accuracy, pitch location, different target, control

## Abstract

Repeated and accurate throwing of an object to a target position is a special human motor skill. It is particularly important to understand accuracy, which has received less attention than speed due to difficulties in measurement. Accuracy has been studied in terms of reducing errors against a single target, but also in terms of distinguishing appropriate throws for targets in different positions. In this study, this ability was investigated by evaluating the two-dimensional distributions of the pitch locations of 15 pitches to three target positions in university students with and without baseball experience. The center, major and minor axis length, major and minor axis ratio, slope, area, and percentage of overlapping area of the 95% confidence ellipse were compared between target positions and participants using a two-way repeated-measures analysis of variance (ANOVA). The center and area of the ellipse indicate the mean and variability of the error, respectively. The lengths of the major axes correspond to the variability of the release timing, and the minor axes correspond to the variability of the release point in space. Therefore, the ratio of the major and minor axes indicates how the variability of the pitching motion is controlled. The slope of the ellipse corresponds to the throwing arm's trajectory, and the percentage of overlap area means the ability to distinguish throws at different target positions. The result showed a main effect of participants on all indices except the center of the ellipse. This indicates that participants can generally distinguish throws by target positions regardless of their baseball experience, although participants with baseball experience may naturally reduce variability. Furthermore, participants with baseball experience demonstrated a decrease variability in release timing, which is a primary contributor to the pitch location variability, relative to the spatial variability of the pitching movements. This reduction in timing variability may be attributed to advanced motor control mechanisms.

## 1. Introduction

Throwing an object at high speed to a target position is a special human motor skill. This can be observed in various sports such as baseball, basketball, and handball. Throwers must achieve high speed and accuracy. However, it is well-known that human-movement variability generally increases as speed intensifies (1). Considering this challenge, it is possible that a motor control mechanism exists, enabling individuals to achieve both speed and accuracy in throwing tasks, as indicated in other tasks (2). Unfortunately, accuracy improvements in throwing have received less attention compared to speed enhancements, primarily due to the complexities involved in measurement (3).

When quantifying the accuracy of a movement, the difference between the actual and target movement outcomes is generally defined as the error. The error was further divided into constant and variable errors, each quantified as the mean and standard deviation (SD) of the error. Because the arrival position at the throw is a two-dimensional quantity in the horizontal and vertical directions, the error is also a two-dimensional quantity. The mean value of the error has components in both the horizontal and vertical directions. In contrast, SD has components in both the horizontal and vertical directions as well as covariance, which is represented by the correlation or direction of the error distribution.

In baseball pitching, accuracy has been studied in terms of error reduction relative to a single target (4, 5). In baseball pitching to a single target, the errors are not evenly distributed vertically and horizontally, but are elliptically distributed (6). Therefore, the mean value and SD of the error can be represented as the center and axis radius (i.e., area) of the error distribution ellipse. The major axis radius of the 95% error distribution ellipse has been reported to be approximately 80 cm for high school pitchers and 60 cm for professional pitchers, and the minor axis radius has been reported to be approximately 50 cm for high school pitchers and 30 cm for professional pitchers in 18.44 m pitching (3).

However, the concept of accuracy encompasses the capacity to differentiate throws for targets at different positions in addition to the ability to minimize errors for a single target. We proposed the concept of “distinguishing the throw” which involves skillfully adjusting the parameters of ball and/or body movement to make the ball reach each target at different spatial position. This ability proves essential in various sports tasks that demand accurate throwing of objects at high speeds to reach specific target positions.

Therefore, this study focused on the ability to appropriately distinguish throws for different target positions. Two-dimensional distributions of the pitch locations of 15 pitches to three target positions in university students with and without baseball experience were evaluated. Because the distribution of pitch locations is affected by throwing arm's trajectory (6), a 95% error distribution ellipse was evaluated to clarify the strategy used to distinguish throws. When a right overarm participant aims at a target, pitch locations along a right-up-left-down ellipse are distributed around the left-low and right-high areas, but are not distributed around the right-low and left-high areas. Therefore, the target positions were set at the center (C), lower left (L), and lower right (R). When a right overarm participant throws at target C, the pitch locations are distributed around target L but not around target R. Therefore, there can be different strategies to distinguish throws according to target (e.g., target C and target L, and target C and target R). If the error distribution ellipses are different for different target positions, it can be concluded that the participants have changed their throwing arm's trajectory. We hypothesized that there would be a difference in the distribution of pitch locations between the level of the participants and the target positions.

## 2. Methods

### 2.1. Experiment

Eleven university students with more than 6 years of baseball experience (gender: male; age: 20.9 ± 1.9 years; height: 174.6 ± 6.9 cm; weight: 71.3 ± 8.5 kg; ten right-handed and one left-handed) and eleven university students without baseball experience (gender: male; age: 24.6 ± 2.4 years; height: 173.2 ± 5.0 cm; weight: 62.4 ± 6.2 kg; ten right-handed and one left-handed) participated in the study. None of the participants had a current injury and their pitching styles were overhand. All experiments were conducted on an outdoor field. Participants warmed up by performing light catching and pitching practice before the experiment. Each participant threw 15 four-seam fastballs at three target positions (0.2 m wide and long) located on a board positioned 18.44 m from the center of the pitcher's plate. The throws were executed in a blocked manner, meaning that each participant threw 15 balls to one target before moving on to the next target. The order of the targets was counterbalanced among the participants. Participants were instructed to aim at the targets and throw them as fast and accurately as possible.

**Figure 1:**
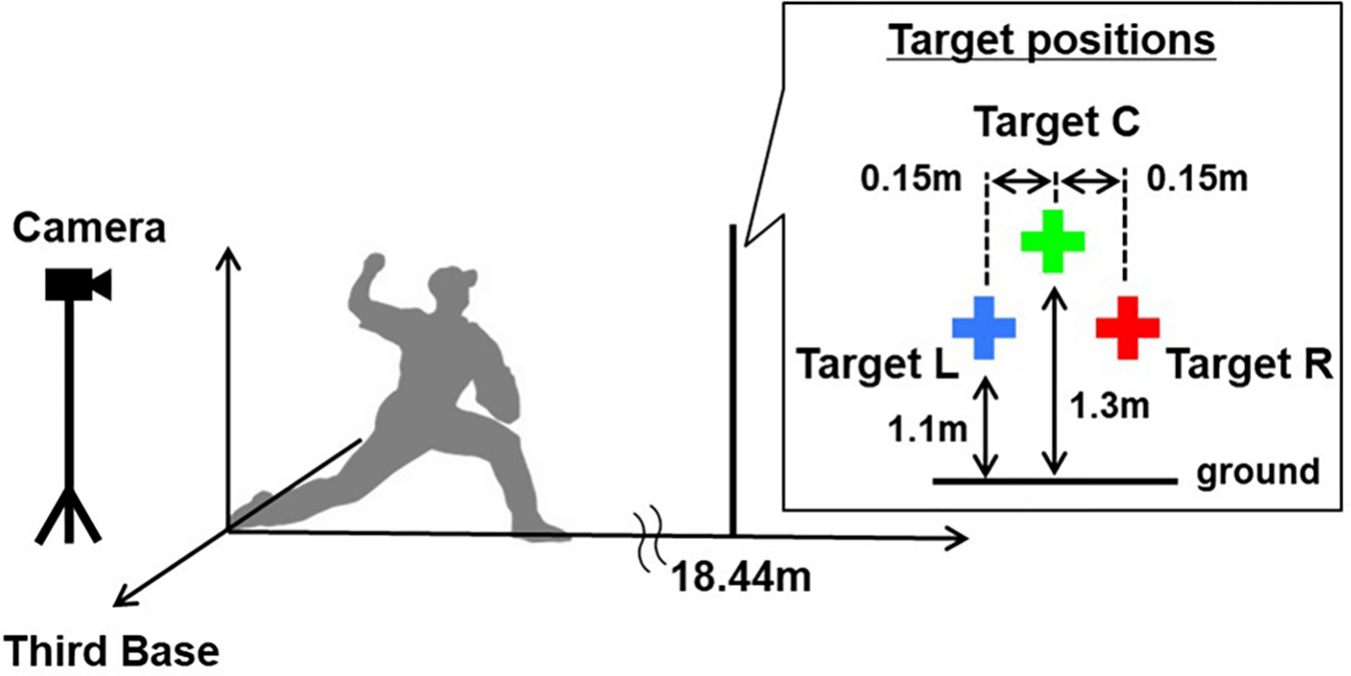
Experimental setup used in this study. Ball movements were recorded using a high-speed camera placed on the participant's back. Three target positions (0.2 m wide and long) were aligned 18.44 m from the center of the pitcher's plate. The center target (C) was located at the center of the pitcher's plate and 1.3 m above the ground, and the left target (L) and right target (R) were aligned 0.15 m to the left and right of target C, respectively and 0.2 m below target C.

The center target (C) was located at the center of the pitcher's plate and 1.3 m above the ground, and the left target (L) and right target (R) were aligned 0.15 m to the left and right of target C, respectively and 0.2 m below target C (Figure 1). This setting was based on the fact that the pitch locations were elliptically distributed, and its slope corresponded to the throwing arm's trajectory. When a right- overarm participant aimed at target C, the pitch locations, which are along a right-up-left-down ellipse, are distributed around target L, but not around target R.

Ball movements were recorded using a high-speed camera (DSC-RX10M4, SONY, Japan; 960 fps) placed on the participant's back (Figure 1). There were no markings on the ball. Consequently, the position coordinates of the center of the ball were determined from the camera images. The motion phase for data collection was approximately 1,000 ms, from the beginning of the throwing motion to approximately 10 ms after the arrival of the ball. The study was approved by the ethics committee of the University of Tokyo, and all participants provided written informed consent.

### 2.2. Data analysis

The position coordinates of the pitch location were obtained using high-speed camera images and numerical analysis software (MATLAB, MathWorks, Japan). The points on the pitch in the high-speed camera images were obtained by digitizing the center point of the ball at the moment of arrival. The moment of arrival was defined as the moment that the ball hit the board, even if it missed a target, since the targets were positioned on the board. To obtain the position coordinates, we calibrated four points in the horizontal direction (at intervals of 2.5, 2.44, 2.5 m) and four points in the vertical direction (at intervals of 0.62, 0.81, 0.77 m), giving a total of 16 calibration points for the transformation of the position coordinates. For the calibration, marks on the board digitized and their measured length was used. The maximum error was confirmed to be 0.01 m. Pitch position coordinates were calculated using a direct linear transformation (DLT).

The two-dimensional distribution of pitch locations for each participant was fitted to a bivariate normal distribution, and a 95% confidence ellipse was calculated (6). The distribution of pitch locations was evaluated using the center, major and minor axis length, the major and minor axis ratio, slope, and area of the 95% confidence ellipse. For left-handed pitchers, the values are presented with the left and right sides inverted. The center and area of the ellipse indicate the mean and variability of the error, respectively. The major and minor axis length of the ellipse indicates the variability of pitch location in each axial direction conceptually. The lengths of the major axes can be considered to correspond to the variability of the release timing, and the minor axes correspond to the variability of the release point in space (6). The “timing” means release timing along the direction of the throwing arm's trajectory and “space” means the release point in space, not aligned with the throwing arm's trajectory. Therefore, timing and space can be divided here. The variability along the major axis is influenced by the variability of the release timing along the direction of the throwing arm's trajectory. On the other hand, that along the minor axis is influenced by the variability of the release point in space, not aligned with the throwing arm's trajectory. Based on this idea, the ratio of the major and minor axes was used as an index to provide how the variability of the pitching motion is controlled. In this study, the minor axis was divided by the major axis. The larger this value, the more the timing variability is reduced to the spatial variability of the pitching motions, resulting in the ellipse resembling a regular circle. The slope of the ellipse corresponds to the throwing arm's trajectory (e.g., right-overarm pitchers pitched along a right-up-left-down ellipse). It was defined as the angle of the major axis, which could range from 0° to 180° (0° means completely horizontal and 90° means a completely vertical major axis). Furthermore, the distance between the target and the center of the 95% confidence ellipse divided to along each axis was calculated. This indicates bias of release timing. If the center of the ellipse was biased toward the upper right along the major axis, this could show difficulty in delaying release timing (holding the ball longer). On the other hand, if the center of the ellipse was biased toward the lower left along the major axis, this could show difficulty in advancing release timing (holding the ball shorter). In addition, to clarify the ability to distinguish throws at different target positions, the percentage of overlap area of the 95% confidence ellipse was evaluated for each target.

The center, major and minor axis length, major and minor axis ratio, slope, area, and percentage of overlap area of the 95% confidence ellipse were compared between the three target positions (L, C, and R) and participants (with and without baseball experience) using a two-way repeated-measures analysis of variance (ANOVA). Statistical significance was defined as *p* < 0.05 throughout the study.

## 3. Results

### 3.1. Two-dimensional distribution of the pitch locations

**Figure 2:**
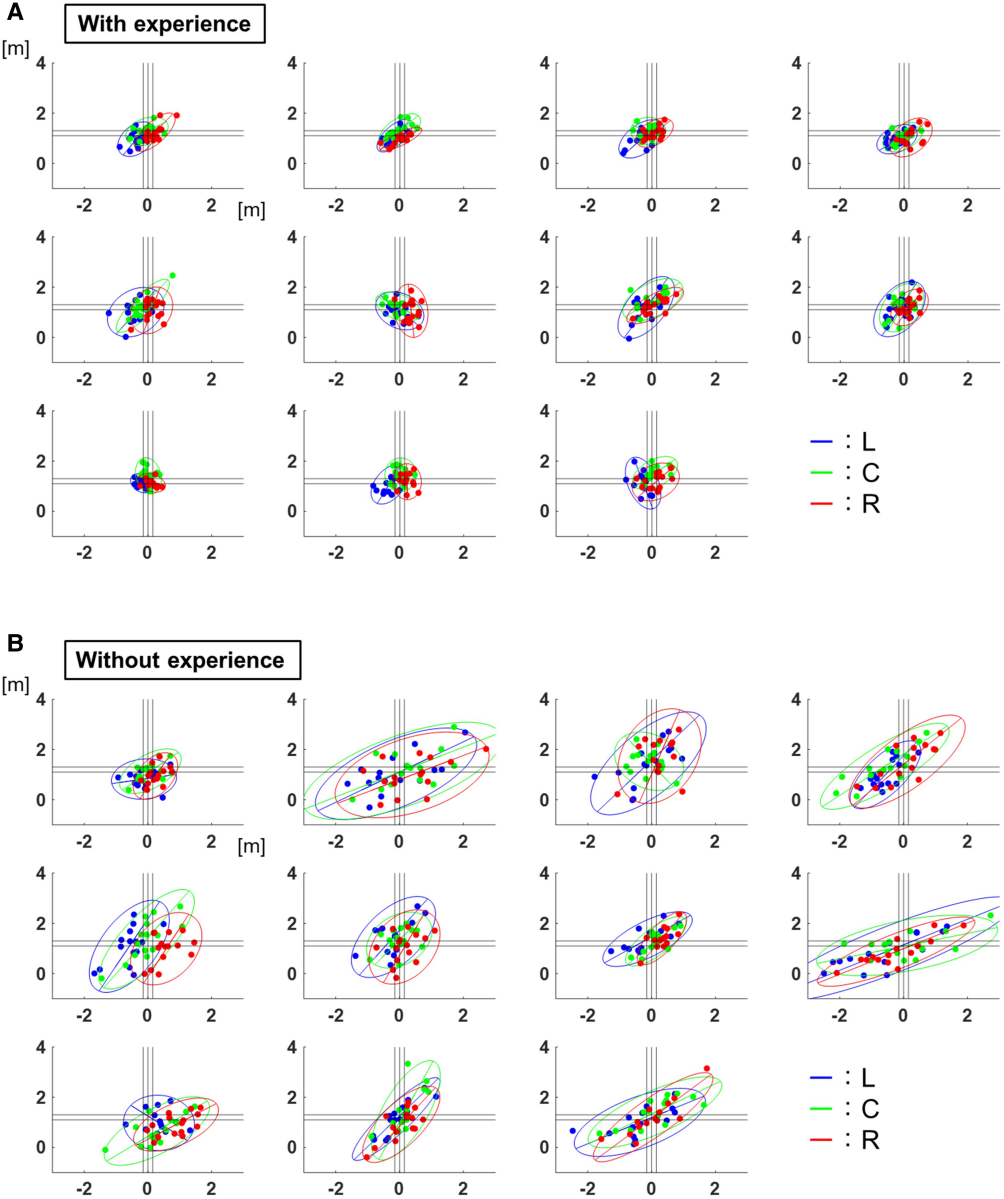
Two-dimensional distribution of the pitch locations. (**A**) The distribution of pitch locations with 95% confidence ellipses for each participant with baseball experience. The lines show 95% confidence ellipses, and the plots show the values of each trial. The blue corresponds to the results of target L, the green corresponds to those of target C, and the red corresponds to those of target R. (**B**) The distribution of the pitch locations with 95% confidence ellipses for each participant without baseball experience. The lines show 95% confidence ellipses, and the plots show the values of each trial. The blue corresponds to the results of target L, the green corresponds to those of target C, and the red corresponds to those of target R.

Figure 2 shows the distribution of pitch locations with 95% confidence ellipses for each participant. For left-handed pitchers, the values are shown with the left and right sides inverted.

### 3.2. The center of the 95% confidence ellipse (mean of error)

**Figure 3:**
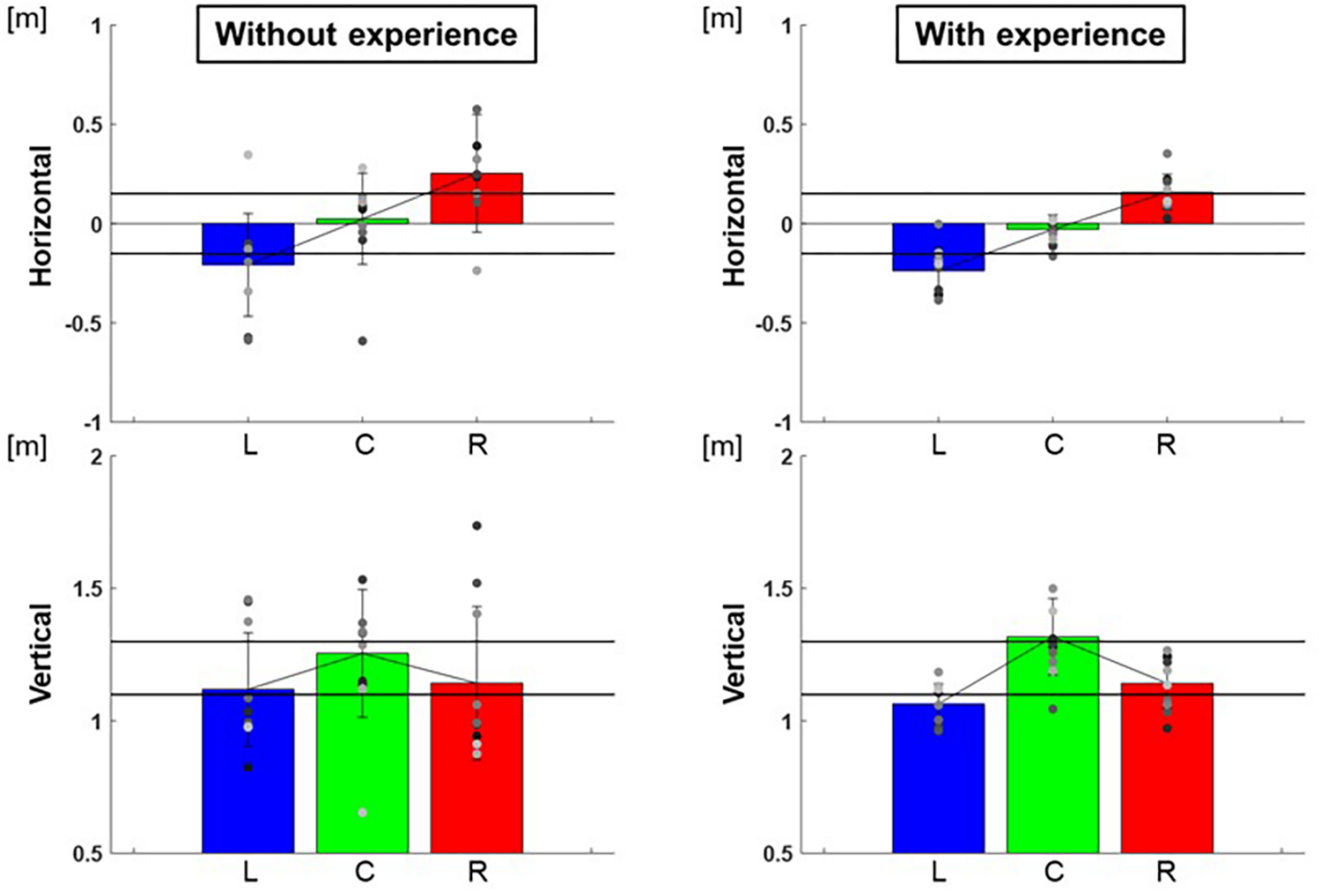
The center of the 95% confidence ellipse. The graphs show the center of the 95% confidence ellipse. The bar graphs show the average of all participants, and the plots show the value for each participant. The error bars show the standard deviations (SD) of all participants. Blue, green, and red correspond to the results for targets L, C, and R, respectively.

**Figure 4:**
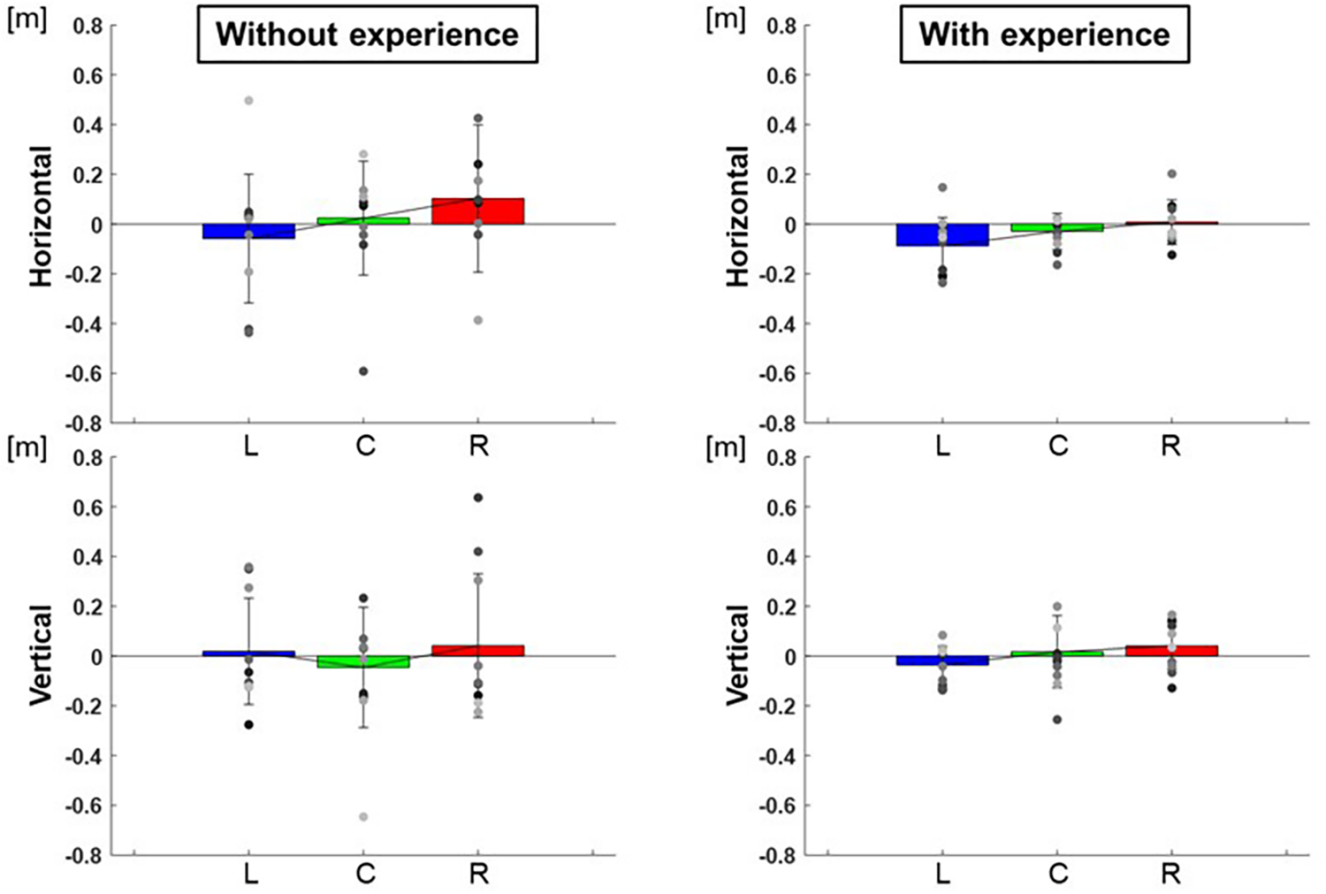
The center of the 95% confidence ellipse (mean of error). The graphs show the center of the 95% confidence ellipse with respect to the targets. The bar graphs show the average of all participants, and the plots show the value for each participant. The error bars show the SD of all participants. Blue, green, and red correspond to the results for targets L, C, and R, respectively.

The center of the ellipse to each target were L: −0.24 ± 0.01 m, C: −0.03 ± 0.01 m, R: 0.16 ± 0.01 m in the horizontal and L:1.06 ± 0.01 m, C: 1.32 ± 0.02 m, R: 1.14 ± 0.01 m in the vertical for participants with baseball experience, and L:−0.21 ± 0.07 m, C: 0.02 ± 0.05 m, R: 0.25 ± 0.09 m in the horizontal and L: 1.12 ± 0.05 m, C: 1.25 ± 0.06 m, R: 1.14 ± 0.08 m in the vertical for participants without baseball experience (Figure 3). These are adjusted to errors with respect to the targets, which are L: −0.09 ± 0.01 m, C: −0.03 ± 0.01 m, R: 0.01 ± 0.01 m in the horizontal and L: −0.04 ± 0.01 m, C: 0.02 ± 0.02 m, R: 0.04 ± 0.01 m in the vertical for participants with baseball experience, and L: −0.06 ± 0.07 m, C: 0.02 ± 0.05 m, R: 0.10 ± 0.09 m in the horizontal and L: 0.02 ± 0.05 m, C: −0.05 ± 0.06 m, R: 0.04 ± 0.08 m in the vertical for participants without baseball experience (Figure 4). Two-way repeated-measures analysis of variance (ANOVA) for the error in the horizontal (target positions: *F*(2,66) = 2.32, *p* = 0.11; participant: *F*(1,66) = 1.49, *p* = 0.23; target positions × participant interaction: *F*(2,66) = 0.16, *p* = 0.86) and vertical (target positions: *F*(2,66) = 0.56, *p* = 0.57; participant: *F*(1,66) = 0.00, *p* = 0.96; target positions × participant interaction: *F*(2,66) = 0.51, *p* = 0.60) showed no main effect of target positions or participants. This indicates that there was no significant difference in the mean of error between the target positions or among the participants.

### 3.3. The length of major and minor axes and the area of the 95% confidence ellipse (variability of error)

**Figure 5:**
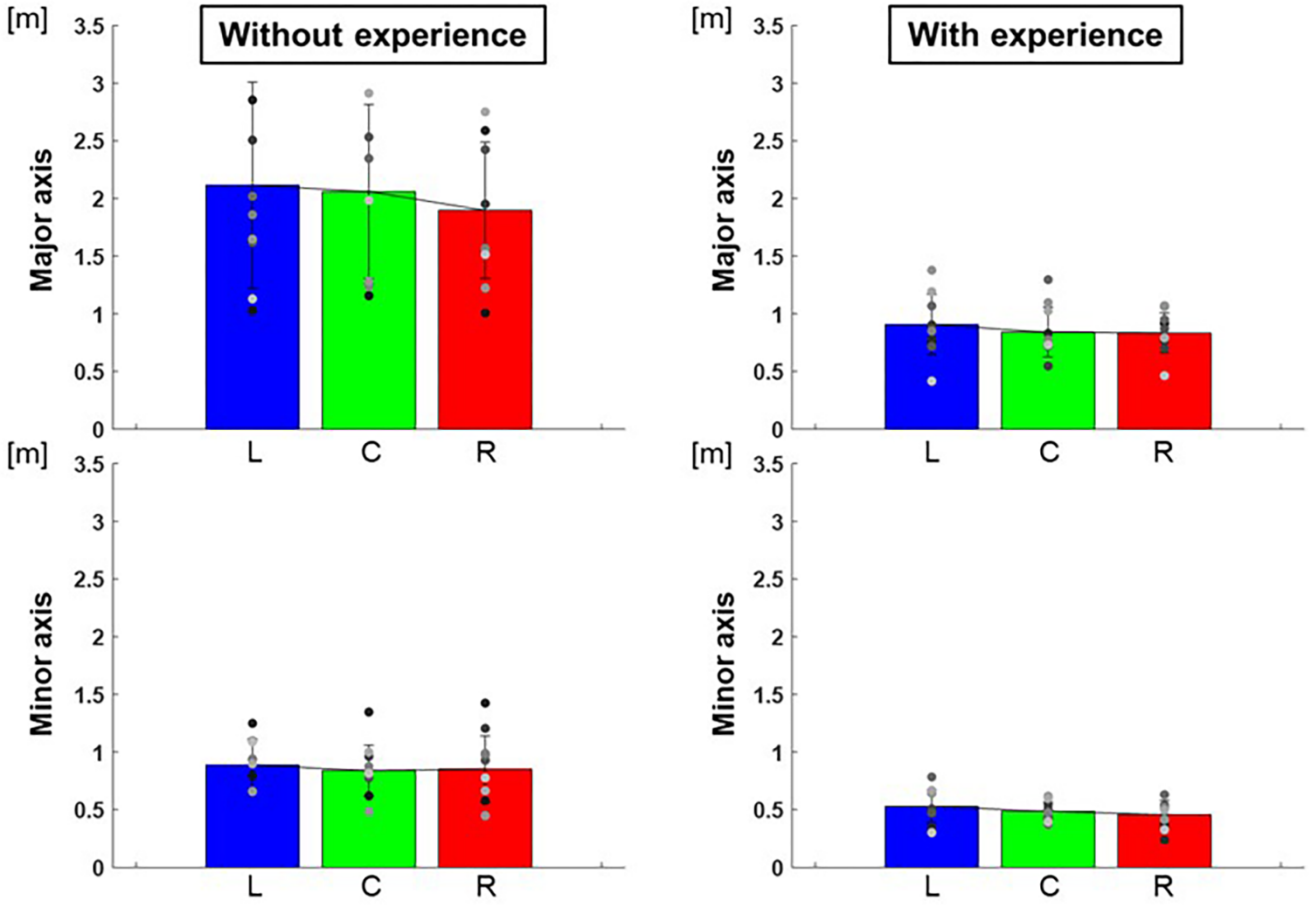
The length of the major and minor axes of the 95% confidence ellipse (variability of error). The graphs show the lengths of the major and minor axes of the 95% confidence ellipses. The bar graphs show the average of all participants, and the plots show the value for each participant. The error bars show the SD of all participants. Blue, green, and red correspond to the results for targets L, C, and R, respectively.

**Figure 6:**
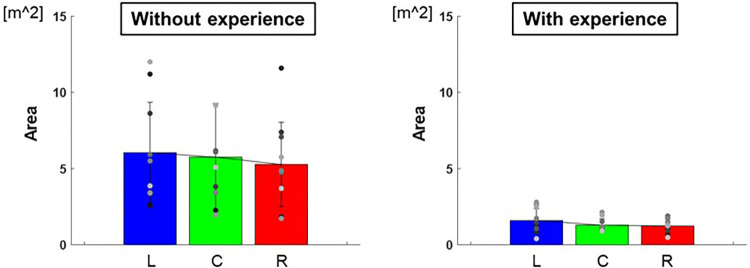
The area of the 95% confidence ellipse (variability of error). The graphs show the areas of the 95% confidence ellipses. The bar graphs show the average of all participants, and the plots show the value for each participant. The error bars show the SD of all participants. Blue, green, and red correspond to the results for targets L, C, and R, respectively.

The length of the major axes of the ellipse to each target were L: 0.91 ± 0.07 m, C: 0.84 ± 0.05 m, R: 0.83 ± 0.03 m and those of the minor axes were L: 0.53 ± 0.02 m, C: 0.49 ± 0.01 m, R: 0.46 ± 0.01 m for participants with baseball experience, and that of major axes were L: 2.12 ± 0.80 m, C: 2.06 ± 0.57 m, R: 1.90 ± 0.35 m and that of minor axes were L: 0.89 ± 0.05 m, C: 0.84 ± 0.05 m, R: 0.85 ± 0.08 m for participants without baseball experience (Figure 5). The area of ellipse to each target were L: 1.59 ± 0.59 m^2^, C: 1.29 ± 0.17 m^2^, R: 1.23 ± 0.21 m^2^ for participants with baseball experience, and L: 6.05 ± 10.92 m^2^, C: 5.75 ± 12.44 m^2^, R: 5.27 ± 7.67 m^2^ for participants without baseball experience (Figure 6). Two-way repeated-measures ANOVA showed a main effect of participants in major axes (target positions: *F*(2,66) = 0.38, *p* = 0.68; participants: *F*(1,66) = 72.02, *p* < 0.01; target positions × participant interaction: F(2,66) = 0.13, *p* = 0.88) and in minor axes (target positions: *F*(2,66) = 0.49, *p* = 0.61; participant: *F*(1,66) = 60.06, *p* < 0.01; target positions × participant interaction: *F*(2,66) = 0.07, *p* = 0.93). Thus, there was a main effect of participants in the areas of the ellipse (target positions: *F*(2,66) = 0.33, *p* = 0.72; participant: *F*(1,66) = 57.75, *p* < 0.01; target positions × participant interaction: *F*(2,66) = 0.06, *p* = 0.94). These results indicate that the variability of error is lower for participants with baseball experience.

### 3.4. The ratio of major and minor axes and the slope of the 95% confidence ellipse (pitching movement and the control its variability)

**Figure 7:**
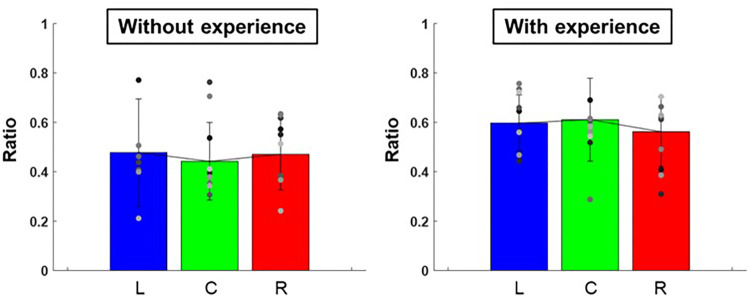
The ratio of major and minor axes of the 95% confidence ellipse (pitching motion and the control of its variability). The graphs show the ratios of the major and minor axes of 95% confidence ellipse. The bar graphs show the average of all participants, and the plots show the value for each participant. The error bars show the SD of all participants. Blue, green, and red correspond to the results for targets L, C, and R, respectively.

**Figure 8:**
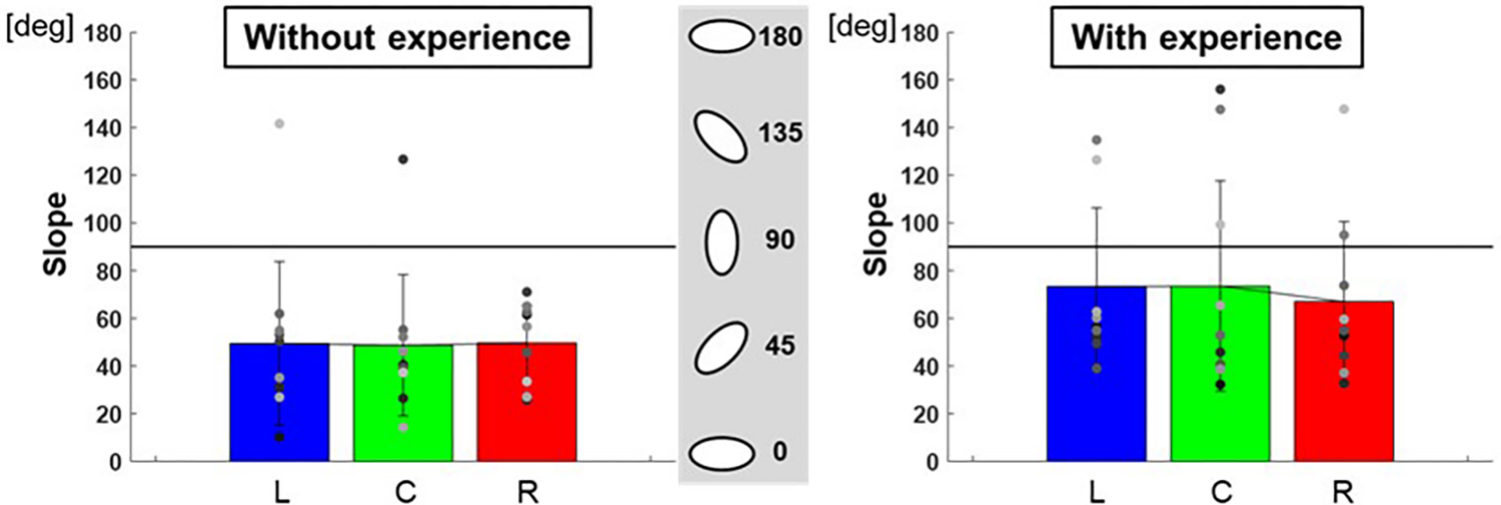
The slope of the 95% confidence ellipse (pitching motion and the control of its variability). The graphs show the slope of the 95% confidence ellipse. The bar graphs show the average of all participants, and the plots show the value for each participant. The error bars show the standard deviations (SD) of all participants. Blue, green, and red correspond to the results for targets L, C, and R, respectively.

The ratio of the major and minor axes of the ellipse to each target was L: 0.60 ± 0.01, C: 0.61 ± 0.03, R: 0.56 ± 0.02 for participants with baseball experience, and L: 0.48 ± 0.05, C: 0.44 ± 0.02, R: 0.47 ± 0.02 for participants without baseball experience (Figure 7). A two-way repeated-measures ANOVA showed a main effect of participants (target positions: *F*(2,66) = 0.10, *p* = 0.91; participants: *F*(1,66) = 10.30, *p* < 0.01; target positions × participant interaction: *F*(2,66) = 0.32, *p* = 0.73). Moreover, the slope of the ellipse to each target was L: 73.3 ± 1,089.37°, C: 73.5 ± 1,947.70°, R: 66.9 ± 1,131.20° for participants with baseball experience, and L: 49.4 ± 1,177.04°, C: 48.7 ± 874.82°, R: 49.6 ± 247.15° for participants without baseball experience (Figure 8). A two-way repeated-measures ANOVA showed a main effect of participants (target positions: *F*(2,66) = 0.06, *p* = 0.94; participants: *F*(1,66) = 7.40, *p* < 0.01; target positions × participant interaction: *F*(2,66) = 0.09, *p* = 0.92). These results indicate that participants with baseball experience possess the ability to control and reduce the timing variability relative to the spatial variability of pitching movements (for more detailed information, refer to the discussion section).

### 3.5. The center of the 95% confidence ellipse divided to along each axis (bias of release timing)

**Figure 9:**
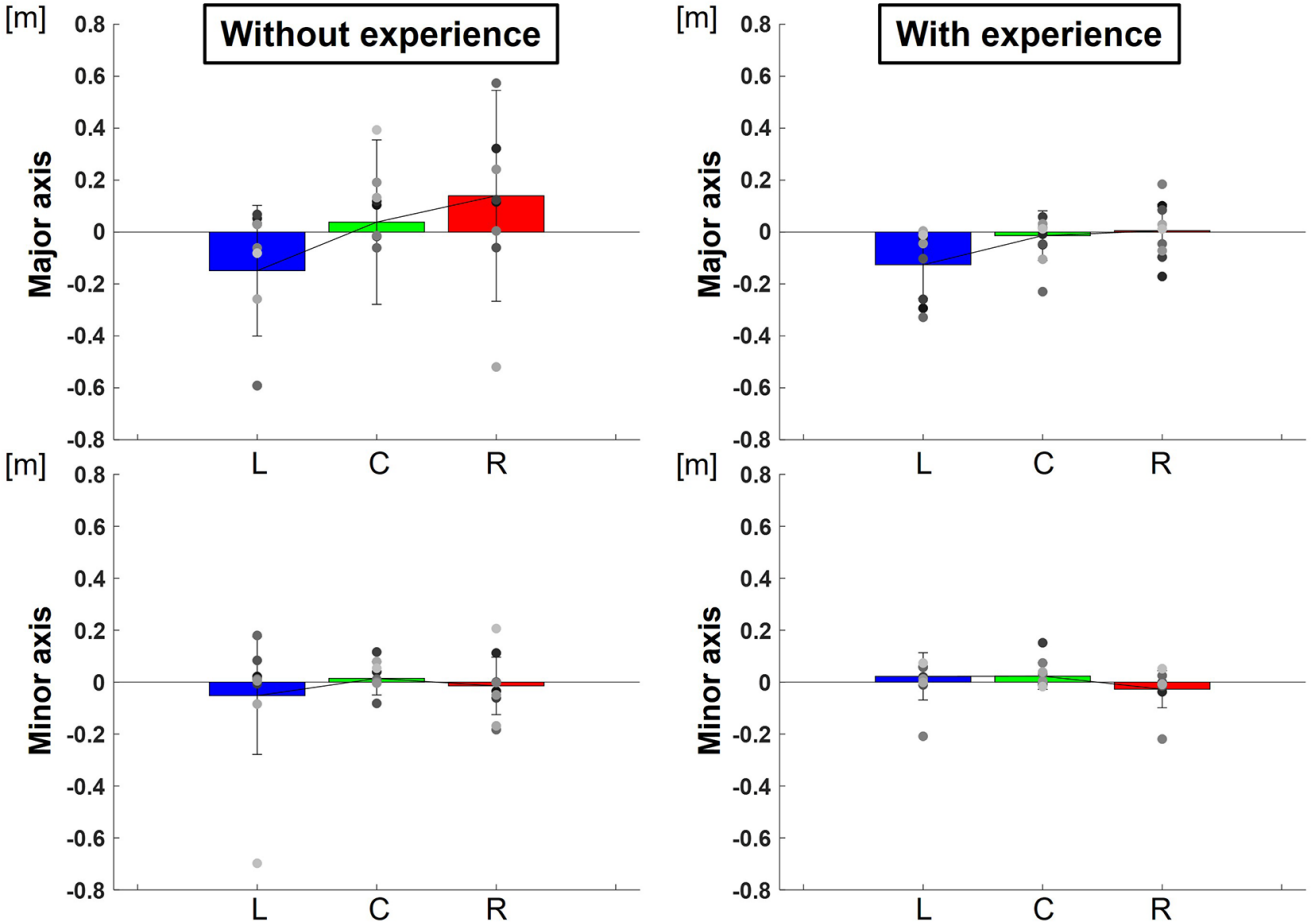
The center of the 95% confidence ellipse divided to along each axis (bias of release timing). The graphs show the center of the 95% confidence ellipse with respect to the targets divided to along each axis. The bar graphs show the average of all participants, and the plots show the value for each participant. The error bars show the SD of all participants. Blue, green, and red correspond to the results for targets L, C, and R, respectively.

The center of the ellipse to each target were L: −0.13 ± 0.02 m, C: −0.01 ± 0.01 m, R: 0.01 ± 0.01 m in major axes and L: 0.02 ± 0.01 m, C: 0.02 ± 0.00 m, R: −0.03 ± 0.01 m in minor axes for participants with baseball experience, and L: −0.15 ± 0.06 m, C: 0.04 ± 0.10 m, R: 0.14 ± 0.16 m in major axes and L: −0.05 ± 0.05 m, C: 0.01 ± 0.00 m, R: −0.01 ± 0.01 m in minor axes for participants without baseball experience (Figure 9). Two-way repeated-measures ANOVA showed a main effect of target positions in major axes (target positions: *F*(2,66) = 4.23, *p* = 0.02; participants: *F*(1,66) = 0.81, *p* = 0.37; target positions × participant interaction: *F*(2,66) = 0.56, *p* = 0.57) but did not show in minor axes (target positions: *F*(2,66) = 0.72, *p* = 0.49; participant: *F*(1,66) = 0.65, *p* = 0.42; target positions × participant interaction: *F*(2,66) = 0.81, *p* = 0.45). The results indicate that the center of the ellipse was biased toward the upper right along the long axis direction in target R but toward the lower left along the long axis direction in target L.

### 3.6. The percentage of overlap area of the 95% confidence ellipse (ability to distinguish throws)

**Figure 10:**
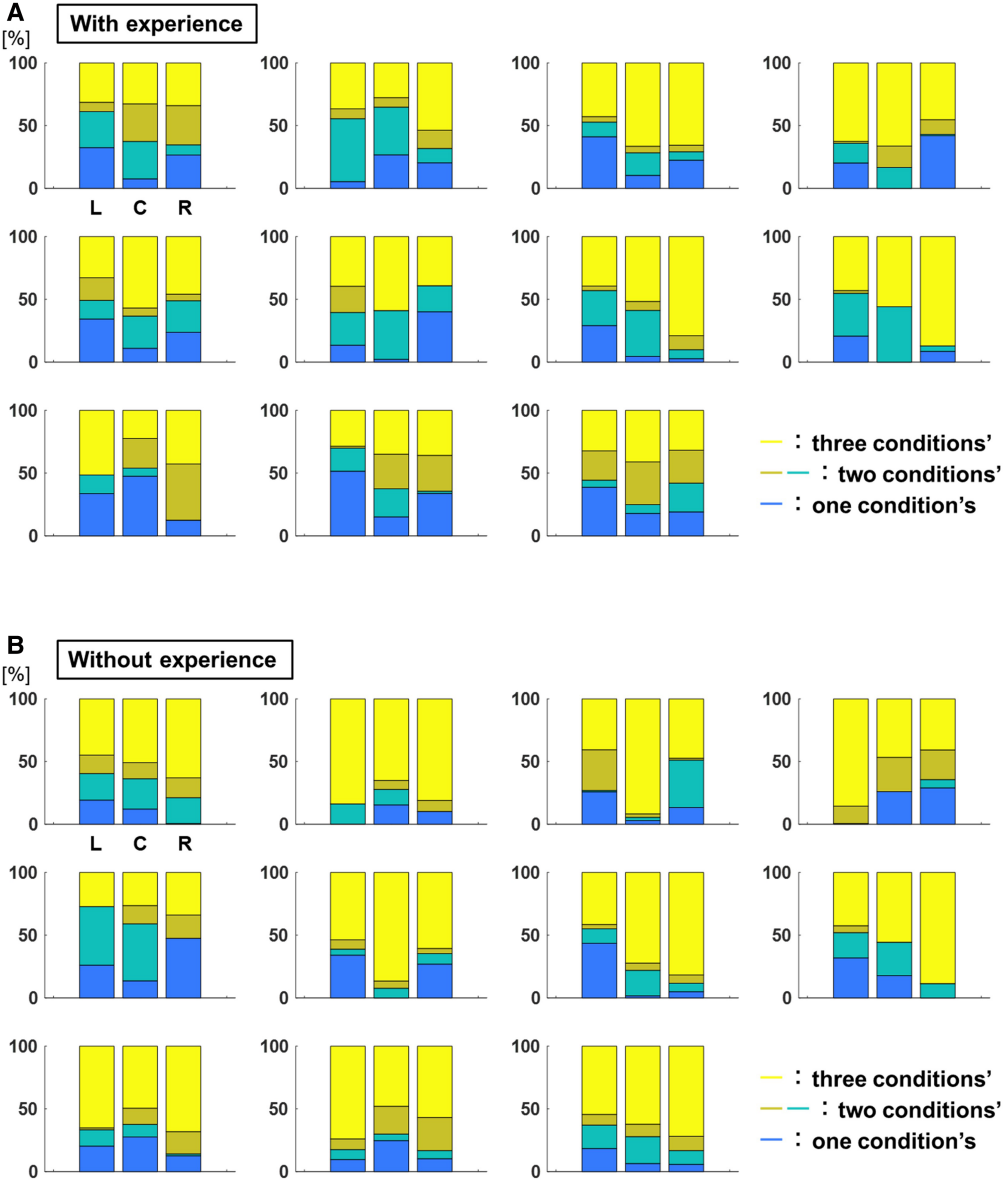
The breakdown of the overlap area of the 95% confidence ellipse (ability to distinguish throws). (**A**) The breakdown of overlap area of 95% confidence ellipses for each participant with baseball experience. The yellow corresponds to the percentage of three conditions’ overlap area, the dark yellow corresponds to that of two conditions’ overlap area, the green corresponds to that of two conditions’ overlap area, and the blue corresponds to that of no overlap area. (**B**) The breakdown of the overlap area of 95% confidence ellipses for each participant without baseball experience. The yellow color corresponds to the percentage of overlap area of three conditions, the dark yellow color corresponds to the overlap area of two conditions, the green color corresponds to the overlap area two conditions, and the blue color corresponds to the no overlap area.

**Figure 11:**
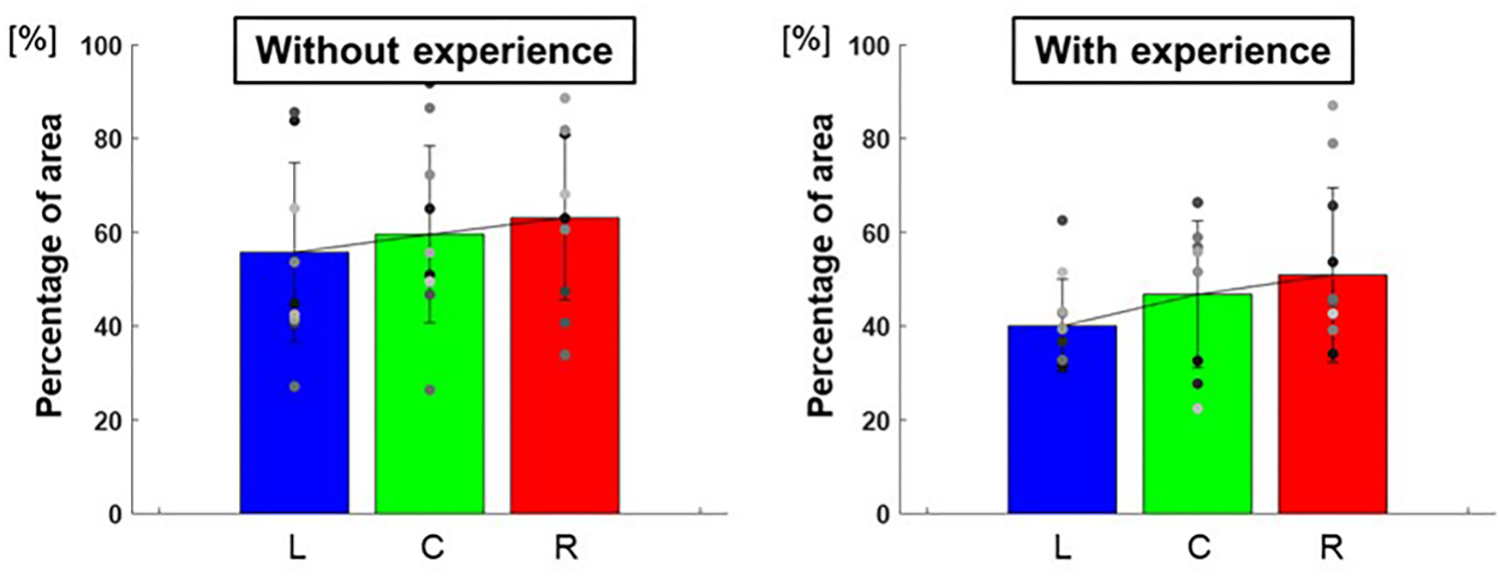
The percentage of the overlap area of the 95% confidence ellipse (ability to distinguish throws). The graphs show the percentage of the overlap area for the three conditions of the 95% confidence ellipse. The bar graphs show the average of all participants, and the plots show the value for each participant. The error bars show the standard deviations (SD) of all participants. Blue, green, and red correspond to the results for targets L, C, and R, respectively.

Figure 10 shows the breakdown of the overlap area of the three conditions, the overlap area of the two conditions, and the area without overlap of the 95% confidence ellipses for each participant. The percentage of the overlap area of the ellipse of the three conditions to each target was L: 40.0 ± 98.7%, C: 46.8 ± 245.5%, R: 50.9 ± 346.7% for participants with baseball experience, and L: 55.7 ± 365.3%, C: 59.6 ± 355.9%, R: 63.1 ± 307.0% for participants without baseball experience (Figure 11). A two-way repeated-measures ANOVA showed a main effect of participants (target positions: *F*(2,66) = 1.60, *p* = 0.21; participants: *F*(1,66) = 10.59, *p* < 0.01; target positions × participant interaction: *F*(2,66) = 0.07, *p* = 0.93). These results indicate that participants with baseball experience had a superior ability to distinguish throws.

## 4. Discussion

The ability to adequately distinguish throws for different target positions on the frontal plane, which we focused on, is important for various motor tasks not only for pitching. Dealing with this ability means trying to answer the question of which parameters to adjust in motor control with redundant degrees of freedom to achieve the goal. However, there are few studies which focused on it despite relevance to these various tasks. This is the first study to focus on the ability to adequately distinguish throws at different target positions during pitching. While previous research has explored dart throwing and rugby throwing with regard to different target distances (7–9), this is the first investigation to specifically focus on the frontal plane in pitching and it is important for these tasks not only for pitching. Furthermore, handball throwing has been the subject of two studies that addressed different target positions, where one study focused a motor learning program and the other explored the speed-accuracy tradeoff for each target (10, 11). However, none of these studies explicitly addressed the ability to adequately distinguish throws. This study examined the two-dimensional distribution of pitch locations at three target positions in participants with and without baseball experience. The center, major and minor axis lengths, major and minor axis ratio, slope, area, and percentage of the overlapping area of the 95% confidence ellipse were compared between the target positions and participants using a two-way repeated-measures ANOVA. The result was that there was a main effect of participants on all indices except the center of the ellipse. Our results can be interpreted as follows.

The center and area of the ellipse indicate the mean and variability of the error, respectively. The result that there was a main effect of participants only on the area of the ellipse means participants can generally distinguish throws by target position regardless of their baseball experience, although the variability of the error was naturally lower for participants with baseball experience. In addition, the length of the major axes corresponds to the variability of the release timing, and the minor axes corresponds to the variability of the release point in space (6), and both were smaller for participants with baseball experience. Although, the reductions in parameters’ variabilities were not always related to the increasing skill level (12), this finding suggests that participants with baseball experience are likely to decrease the variability of their pitching movements both in terms of timing and spatial accuracy.

However, there was a main effect of participants on the ratio of major and minor axes, suggesting how to control the variability of pitching movements. For participants with baseball experience, the ratio was larger i.e., the ellipse was more like a regular circle. This means that participants with baseball experience control and reduce the timing variability to the spatial variability of pitching movements. Previous studies have reported different sources of variability in pitch location in terms of joint kinematics, joint torques, and ball release parameters (13–15). One study reported that the most important source of variability was the timing of ball release (16). The participants with baseball experience demonstrated an ability to decrease this variability in timing, which, in turn, contributed to reducing variability in pitch location. In contrast, a separate study focusing on throwing to targets positioned at different heights and distances in the sagittal plane (from a sitting position) observed that the throws were distinguished based on diverse hand trajectories rather than the timing of the ball release (17). This indicates that the strategies for achieving accuracy in throwing may differ depending on the specific target plane and/or type of throwing task involved. This is relevant to motor control and sports in general.

This may be related to the slope of the ellipse. The slope of the ellipse corresponds to the pitching motion. Therefore, right overarm pitchers pitch along the right-up-left-down ellipse. However, for participants with baseball experience, the slope was greater, and for some even greater than 90°, i.e., a left-up-right-down ellipse. The slope, which does not correspond to pitching motions in participants with baseball experience, may be attributed to the reduction of timing variability relative to spatial variability in pitching motions and the ellipse to resemble a regular circle. If the ellipse becomes a regular circle, concept of major/minor axes and slope no longer exists. Therefore, slope of the major axis is affected by the several outliners and can be of various values when the ellipse resembles a regular circle. Nonetheless, it remains possible that pitching motions still vary between participants with and without baseball experience.

Furthermore, the distance between the target and the center of the 95% confidence ellipse divided to along each axis was calculated. This indicates bias of release timing. The results indicate that the center of the ellipse was biased toward the upper right along the long axis direction in target R but toward the lower left along the long axis direction in target L. This could show difficulty in delaying release timing (holding the ball longer) in target R but advancing timing (holding the ball shorter) in target L.

The percentage of the overlapping area of the 95% confidence ellipse corresponds to the ability to distinguish pitches to different target positions. The percentage of the overlapping area of the three conditions of the ellipse was smaller for participants with baseball experience. This indicates that the participants with baseball experience had a better ability to distinguish throws to different target positions. However, the values were relatively high, 40%–50%, and it is difficult to conclude that they were sufficient to distinguish throws.

Contrary to our hypothesis, there was no main effect of target position on any of the indices. The target setting was based on the pitch location distribution, the slope of which corresponded to the throwing arm's trajectory. When a right overarm participant throws at target C, the pitch locations, which are along a right-up-left-down ellipse, are distributed around target L but not around target R. Therefore, there can be different strategies to distinguish throws according to target (e.g., target C and target L, and target C and target R). The reason why there was no such difference in this study was the difficulty of the task and the level of the participants. For the participants in this study (especially those without baseball experience), the distance between targets, which was 0.15 m in the horizontal direction and 0.2 m in the vertical direction, may have been too small. In fact, the percentage of overlapping area of the three conditions of the ellipse was relatively high, even for participants with baseball experience. It is difficult to conclude that it was sufficient for distinguishing throws. Therefore, further studies with tasks of different difficulty levels and participants of different ability levels are needed to clarify this issue.

This study had some limitations. Only the pitch location was analyzed, and release parameters and physical movements were not considered. For example, the velocity angle of the ball at release (release angle), which is the only factor to determine variability of pitch location (18), could potentially differ depending on the targets being aimed at it. Furthermore, the velocity could potentially affect the variability of pitch location, as throws at different speeds by the same participant may exhibit difference in the accuracy. However, as participants were instructed to throw at their maximum speed while maintaining accuracy and they should actually do that, the difference in speed between participants with and without baseball experience was considered not to affect our results. Participants always threw on the plane ground (not on the mound) and there is no catcher or batter, which is different from the environment in actual baseball games. Participants only threw fifteen 4-seam fastballs aimed at a target located 1.1–1.3 m above the ground, whereas pitchers in real baseball games threw more different types of pitches, including breaking balls, aimed at different corners. A limited number of trials may influence the indexes of the 95% confidence ellipse. Participants with baseball experience were limited to university baseball players (and some of them were not pitchers). On the other hand, there was a broad range of participants without baseball experience. All of them have no baseball experience but various other sports experience, which might cause the high variability in the performance. In addition, the pitching motion was specific to the overarm-throwing style. Future research is needed with more participants of varying abilities and pitching styles in a more practical setting. Furthermore, this study was based on an idea that the lengths of the major axes correspond to the variability of the release timing, and the minor axes correspond to the variability of the release point in space (6). However, there is no study that provided evidence of a relationship between the axes of the ellipse and the timing or spatial variability of the pitching motion. In order to provide evidence of it, it is possible to create a model of calculating pitch location from the pitching motion and to simulate the changes in pitch location when the timing or spatial variability of the pitching motion vary, for example.

## 5. Conclusion

This study focused on the ability to appropriately distinguish throws for different target positions. Two-dimensional distributions of the pitch locations of 15 pitches to three target positions using university students with and without baseball experience were evaluated. The center, major and minor axis length, major and minor axis ratios, slope, area, and percentage of the overlapping area of the 95% confidence ellipse were compared between the target positions and participants using a two-way repeated-measures ANOVA. The result showed a main effect of participants on all indices except for the center of the ellipse. This indicates that participants can generally distinguish throws by target positions regardless of their baseball experience, although participants with baseball experience may naturally reduce variability. Moreover, participants with baseball experience may control and reduce the timing variability of ball release, which is the most significant source of variability, to the spatial variability of pitching movements.

## Data Availability

The original contributions presented in the study are included in the article/**Supplementary Material**, further inquiries can be directed to the corresponding author.
