# Supplementary material for: The ability to appropriately distinguish throws for different target positions
Source: Front Sports Act Living. 2023 Sep 6;5:1250938. doi: 10.3389/fspor.2023.1250938 (PMC10511760; doi:10.3389/fspor.2023.1250938)
Supplement: Supplementary file 1 [file Table1.docx]

Supplementary Material

The ability to appropriately distinguish throws for different target positions

Ayane Kusafuka*, Rintaro Yamamoto, Taishi Okegawa, Kazutoshi Kudo

*** Correspondence:** Ayane Kusafuka: ayanekusafuka@gmail.com

# The intraclass correlation coefficient (ICC)

The intraclass correlation coefficient (ICC) was performed as reliability statistical tools. ICC(1,15) of pitch location was calculated for each target for all participants (Table 1), participants with experience (Table 2), and without experience (Table 3), respectively. Furthermore, ICC(1,3) of each index of ellipse was calculated for all participants, participants with experience, and without experience (Table 4). In this study, the ICC values differed depending on the targets and indexes. Since the number of trials in this study was 15 for each target, a small number of outliers could affect these values. This point needs to be confirmed by increasing the number of trials in the future.

Table 1 ICC(1,15) of pitch location for all participants.

| Index | L | C | R |
| --- | --- | --- | --- |
| Horizontal | 0.42 | 0.26 | 0.64 |
| Vertical | 0.29 | 0.61 | 0.64 |

Table 2 ICC(1,15) of pitch location for participants with experience.

| Index | L | C | R |
| --- | --- | --- | --- |
| Horizontal | 0.60 | 0.05 | 0.28 |
| Vertical | -0.38 | 0.72 | 0.23 |

Table 3 ICC(1,15) of pitch location for participants without experience.

| Index | L | C | R |
| --- | --- | --- | --- |
| Horizontal | 0.41 | 0.30 | 0.69 |
| Vertical | 0.39 | 0.58 | 0.70 |

Table 4 ICC(1,3) of each index of ellipse.

| Index | All | With experience | Without experience |
| --- | --- | --- | --- |
| Center in horizontal | 0.64 | 0.25 | 0.68 |
| Center in vertical | 0.72 | 0.63 | 0.75 |
| Length of major axis | 0.94 | 0.80 | 0.86 |
| Length of minor axis | 0.93 | 0.60 | 0.89 |
| Area | 0.95 | 0.78 | 0.90 |
| Ratio of two axes | 0.63 | 0.07 | 0.70 |
| Slope | 0.71 | 0.74 | 0.42 |
| Percentage of overlap | 0.54 | 0.52 | -0.90 |
